# Clinical Characteristics of Intraocular Lens Dislocation in Chinese Han Populations

**DOI:** 10.1155/2020/8053941

**Published:** 2020-04-26

**Authors:** Qi Fan, Xiaoyan Han, Xiangjia Zhu, Lei Cai, Xiaodi Qiu, Yi Lu, Jin Yang

**Affiliations:** ^1^Department of Ophthalmology and the Eye Institute, Eye and Ear, Nose, and Throat Hospital, Fudan University, Shanghai, China; ^2^The Key Laboratory of Myopia, Ministry of Health, Shanghai, China; ^3^Shanghai Key Laboratory of Visual Impairment and Restoration, Shanghai, China; ^4^Key National Health Committee of the Key Laboratory of Myopia, Fudan University, Shanghai, China; ^5^The Key Laboratory of Myopia, Chinese Academy of Medical Sciences, Shanghai, China

## Abstract

**Purpose:**

To investigate the clinical characteristics of patients with intraocular lens (IOL) dislocation after IOL implantation in Chinese Han populations.

**Methods:**

The medical records of patients with IOL dislocation were retrospectively reviewed from January 2007 to December 2017, and a total of 312 patients (male: 231, female: 97) (328 eyes) were included in this study. The axial length (AL), IOL power, and the time interval between cataract surgery and IOL dislocation as well as the ocular conditions associated with IOL dislocation were recorded. The IOL dislocation was classified and graded based on its relationship with the capsule and the position of the dislocated IOL.

**Results:**

The mean time between original cataract surgery and IOL dislocation was 5.63 ± 5.13 years; IOL dislocation occurred in up to 56.1% (184 eyes) of the eyes within 5 years. Trauma was found in 136 eyes (41.5%); pars plana vitrectomies were performed in 61 eyes (18.6%), and high myopia was detected in 108 eyes (32.9%). A total of 243 eyes (74.1%) had out-of-the-bag IOL dislocations, while 85 eyes (25.9%) had in-the-bag IOL dislocations. There was a statistically significant difference in the constituent ratio of trauma between in-the-bag dislocation and out-of-the-bag dislocation (Pearson's chi^2^ = 33.3992, *P* < 0.001); ocular blunt traumas were significantly higher in in-the-bag dislocations, while open-globe injuries were significantly higher in out-of-the-bag dislocations. A statistically significant difference was found for the ratio of patients with AL longer than 30 mm between in-the-bag dislocation and out-of-the-bag dislocation (Pearson's chi^2^ = 9.7355, *P* < 0.002).

**Conclusions:**

In Chinese Han populations, the most common IOL dislocation is out-of-the-bag dislocation; the most common risk factors were trauma, long axial length, and eyes undergoing pars plana vitrectomy; a minimum follow-up of 5 years is suggested for IOL dislocation-predisposed eyes undergoing cataract surgery.

## 1. Introduction

Dislocation of an intraocular lens (IOL) is uncommon but is one of the most serious complications following cataract surgery [1]. It is a cause of blindness throughout the world, with an incidence of 0.05–3% [2–4] and a cumulative risk of 0.1% after 10 years and 1.7% after 25 years following cataract surgery [5]. With recent developments in surgical techniques and devices, cataract surgery has now provided a higher safety for patients, though it does not parallel a declined frequency of IOL dislocation due to the increasing number of cataract surgeries [6].

The causes of IOL dislocation include the absence of adequate capsular or zonular support. Previously reported factors associated with zonular weakness or dehiscence and capsular insufficiency were indicated in eyes with pseudoexfoliation syndrome [7], uveitis [8], retinitis pigmentosa [9], previous vitreoretinal surgery [10], increased axial length [6], Marfan syndrome, and trauma [11]. Detailed information on the clinical characteristics of IOL dislocation has been reported in populations such as American, German, Australian, and Swedish populations, among others. However, to the best of our knowledge, little is known about the risk factors for IOL dislocation in Chinese Han populations.

In this study, a retrospective chart review of consecutive patients with IOL dislocation was performed, and the clinical characteristics of IOL dislocation patients in Chinese Han populations were demonstrated and analyzed.

## 2. Materials and Methods

We retrospectively reviewed all charts of subjects with IOL dislocation after IOL implantation surgery between January 2007 and December 2017 in Shanghai, and those who required further surgical intervention were included. This study adhered to the Declaration of Helsinki and was approved by the Ethics Committee of the Eye & ENT Hospital of Fudan University.

Basic information, such as age and sex, axial length (AL), intraocular pressure (IOP), corneal endothelial cell density, corneal astigmatism, time for cataract surgery, time between IOL dislocation and surgical intervention, duration of IOL dislocation, and surgical choices for IOL dislocation, was acquired and demonstrated. Associated ocular conditions when the patients presented to the clinic were also investigated.

### 2.1. Classification of IOL Dislocation

Several standards for classifying IOL dislocation have been established [12–15].

Based on whether or not the dislocated IOL is in the capsule, it can be classified into two categories: in-the-bag dislocation and out-of-the-bag dislocation; it can also be classified as dislocation in the anterior chamber, in the posterior chamber and/or in the anterior vitreous cavity, and in the deep vitreous cavity, relying on the sites of dislocated IOL. For those whose IOL dislocated in the posterior chamber and/or the anterior vitreous cavity, the sites of dislocated IOL were further described as superior, superonasal, nasal, inferonasal, inferior, inferotemporal, and superotemporal, and the severities were graded as mild, moderate, and severe.

### 2.2. Statistical Analysis

SPSS software (SPSS, Inc.) was used for the statistical analysis. Continuous variables are presented as means ± standard deviations. Discrete variables were presented as percentages. Fisher's exact test and chi-squared test were used to analyze the discrete variables. Any differences with *P* values less than 0.05 were considered statistically significant.

## 3. Results

### 3.1. General Clinical Characteristics

**Figure 1:**
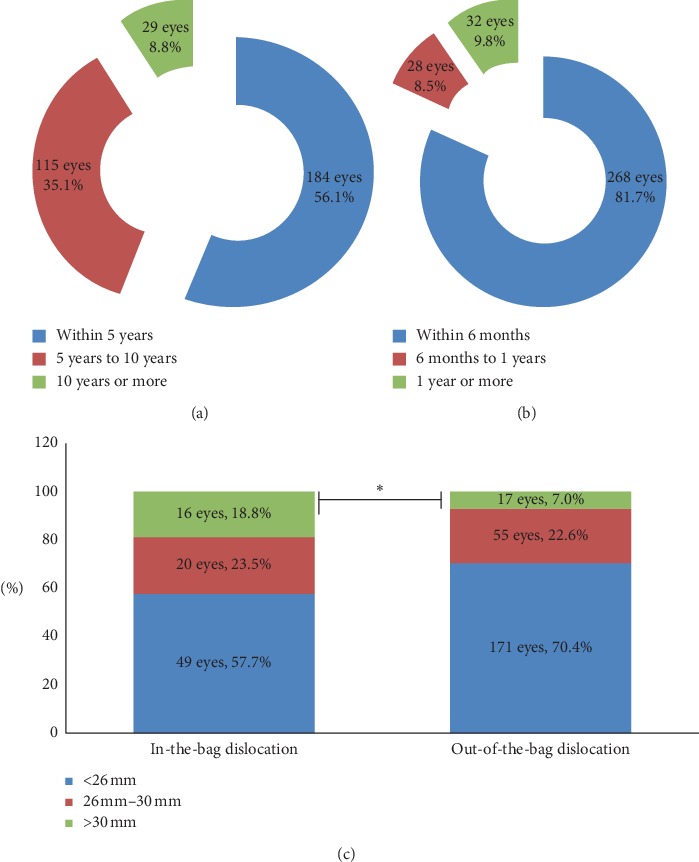
The proportion ratios of time from cataract surgery to IOL dislocation (a), from IOL dislocation to surgical intervention (b), and axial length (c). ^*∗*^Statistically significant differences between in-the-bag dislocation and out-of-the-bag dislocation were found for the ratio of patients with AL longer than 30 mm.

A total of 312 patients (male: 231, female: 97) (328 eyes, 16 patients had bilateral IOL dislocations) were included in this study, with a mean age of 48.10 ± 20.17 years, ranging from 2 to 85 years (Table 1). The included patients had an average time from cataract surgery to the onset of IOL dislocation of 5.63 ± 5.13 years (1 day to 23.39 years, Table 1), with 56.1% (184 eyes) occurring within 5 years, 35.1% (115 eyes) between 5 and 10 years, and 8.8% (29 eyes) in more than 10 years (Figure 1(a)). The mean time to surgical intervention was 0.53 ± 1.40 years (1 day to 15.39 years), with 81.7% (268 eyes) of the patients within 6 months, 8.5% (28 eyes) between 6 months and 12 months, and 9.8% (32 eyes) in more than one year (Figure 1(b)).

The mean AL was 25.65 ± 3.31 mm (17.74 to 36.69 mm). For in-the-bag dislocations, the mean AL was 26.51 ± 3.78 mm (20.86 to 35.86 mm), with 18.8% (16 eyes) of patients having an AL longer than 30 mm, 23.5% (20 eyes) between 26.0 mm and 30.0 mm, and 57.6% (49 eyes) shorter than 26 mm. For out-of-the-bag dislocations, the mean AL was 25.34 ± 3.07 mm (17.74 to 36.69 mm), with 7.0% (17 eyes) of patients having an AL longer than 30 mm, 22.6% (55 eyes) between 26.0 mm and 30.0 mm, and 70.4% (171 eyes) shorter than 26 mm (Figure 1(c)). Statistically significant differences between in-the-bag dislocation and out-of-the-bag dislocation were found for the ratio of patients with AL longer than 30 mm (Pearson's chi^2^ = 9.7355, *P* value = 0.002).

### 3.2. Associated Ocular Conditions

Associated ocular conditions with IOL dislocation are shown in Table 2. A total of 168 eyes (51.2%) had more than one associated ocular condition.

A total of 136 eyes (41.5%) were associated with trauma. Of these, 95 eyes (29.0%) were open-globe injuries, and 41 eyes (12.5%) were ocular blunt trauma. A statistically significant difference between the constituent ratio of trauma before and after primary IOL implantation was found (Pearson's chi^2^ = 100.25, *P* value < 0.001). The ratio of open-globe injuries was significantly higher before primary IOL implantation than after primary IOL implantation, while ocular blunt trauma was significantly higher after primary IOL implantation than before primary IOL implantation.

A total of 172 eyes (52.4%) had ocular diseases (Table 2). High myopia was detected in 108 eyes (32.9%) (AL was longer than 26.00 mm); preexisting lens subluxation before original cataract surgery appeared in 32 eyes (9.8%). Other associated ocular diseases were as follows: retinitis pigmentosa (15 eyes/4.6%), chronic uveitis (6 eyes/1.8%), glaucoma (5 eyes/1.5%), iris cyst (4 eyes/1.2%), and ocular siderosis (2 eyes/0.6%).

A total of 108 eyes (32.9%) had ocular surgeries conditions (Table 2). Pars plana vitrectomy was performed in 61 eyes (18.6%), and causes of vitrectomy included retinal detachment (22 eyes/36.1%), traumatic vitreoretinopathy (19 eyes/31.1%), intraocular foreign body (10 eyes/16.4%), macular hole (5 eyes/8.2%), diabetic retinopathy (3 eyes/4.9%), and endophthalmitis (2 eyes/3.3%). A total of 30 eyes (9.1%) underwent surgery for congenital cataract. Other associated surgeries were as follows: trabeculectomy (6 eyes/1.8%), strabismus correction (6 eyes/1.8%), scleral buckling (3 eyes/0.9%), and penetration keratoplasty (2 eyes/0.6%).

A total of 42 eyes (12.8%) had a history of capsule rupture during cataract surgery; 8 eyes (2.4%) received Nd : YAG laser capsulotomy, and 21 eyes (6.4%) had capsular contraction syndrome.

### 3.3. Dislocation Sites and Grading

Detailed information on IOL dislocation classified by the aforementioned classification standard is provided in Table 3.

**Figure 2:**
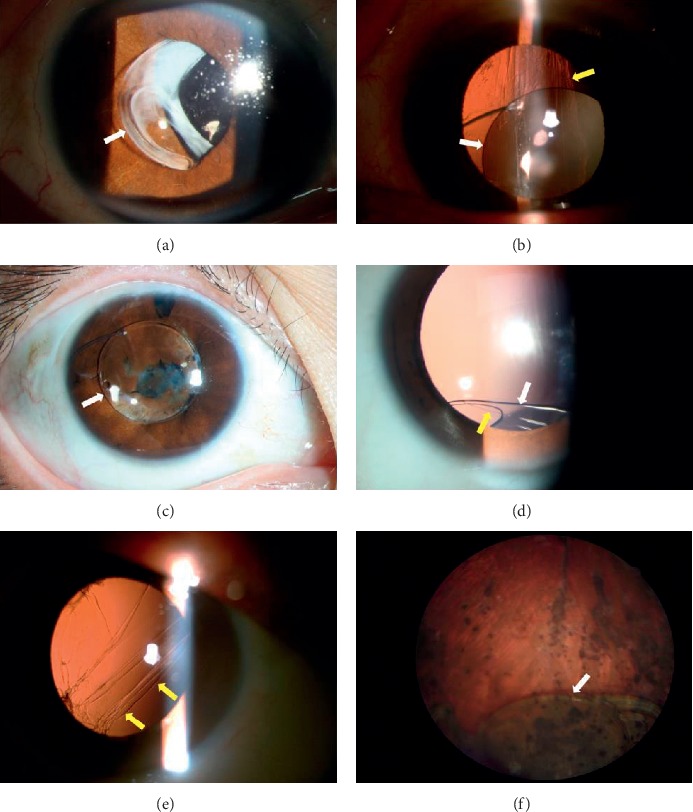
Representative photographs of IOL dislocation. (a) Capsular bag complex dislocation; (b) out-of-the-bag dislocation; (c) prolapse into the anterior chamber; (d) prolapse into the anterior vitreous cavity; (e) and (f) (e) presents the ruptured capsule and absence of IOL, while (f) presents IOL dislocated completely into the vitreous. White arrows show dislocated IOL, while yellow arrows require a ruptured capsule.

A total of 85 eyes (25.9%) were seen with in-the-bag dislocations (Figure 2(a)), and 243 eyes (74.1%) had out-of-the-bag dislocations (Figure 2(b)). IOL dislocation sites were as follows: the intraocular lens was dislocated into the anterior chamber in 39 eyes (11.9%) (Figure 2(c)), into the posterior chamber and/or the anterior vitreous cavity in 184 eyes (56.1%) (Figure 2(d)), and completely into the vitreous in 105 eyes (32.0%) (Figures 2(e) and 2(f)). The major dislocation sites were inferior in 121 eyes (65.8%) (Table 4).

### 3.4. In-the-Bag Dislocations versus Out-of-the-Bag Dislocations

Associated ocular conditions with in-the-bag and out-of-the-bag dislocations are shown in Table 5.

The most common associated ocular conditions with in-the-bag dislocations were high myopia in 36 eyes (42.4%), trauma in 26 eyes (30.6%), and retinitis pigmentosa in 13 eyes (15.3%), while the most common associated ocular conditions with out-of-the-bag dislocations were trauma in 110 eyes (45.3%) (especially open-globe injuries), high myopia in 72 eyes (29.6%), and pars plana vitrectomy in 50 eyes (20.6%). There was a statistically significant difference between in-the-bag dislocation and out-of-the-bag dislocation on the constituent ratio of trauma (Pearson's chi^2^ = 33.3992, *P* < 0.001), and ocular blunt traumas were significantly higher in in-the-bag dislocations, while open-globe injuries were significantly higher in out-of-the-bag dislocations.

### 3.5. Surgical Procedures for IOL Dislocation

Surgical techniques to treat IOL dislocation included IOL exchange, reposition, refixation, and explantation (Table 6). A total of 19 eyes (5.8%) suffered from two instances of IOL repositioning. IOL exchange and refixation were the most common surgical procedures, accounting for 39.9% (131 eyes) and 25.9% (85 eyes), respectively. Sutured scleral fixation is most commonly used for both IOL exchange (98 eyes/29.9%) and refixation (78 eyes/23.8%).

## 4. Discussion

As cataract patients are increasing with the aging of the population, IOL dislocation is an unusual but not rare complication following cataract surgery, even with noted progress in surgical techniques and devices. According to this study, the predisposing factors may differ due to ethnic and geographical differences. To the best of our knowledge, the characteristics of IOL dislocation in the Chinese Han population are seldom reported. Due to the surgical difficulty involved in IOL dislocation, patients in eastern China are concentrated in Shanghai to deal with IOL dislocation. As a tertiary referral clinic area and one of the most important ophthalmic centers in China, we have relatively easy access to a larger amount of data relating to the IOL dislocation, based on which its clinical characteristics are provided in this study so that they may further assist in managing this complication in practice.

Contrary to the previous reports, the majority of which have focused on in-the-bag dislocation, our study has demonstrated the clinical characteristics of both in-the-bag and out-of-the-bag dislocation. In light of other ethnic studies [3], pseudoexfoliation was referred to as the most common risk factor predisposing IOL to dislocate, followed by other ocular conditions, such as uveitis, trauma, vitrectomy, and increased axial length, which disagrees with the findings in our study that the most common risk factors were trauma, high myopia, and eyes undergoing pars plana vitrectomy in a Chinese population. This finding may be related to the high incidence of high myopia and the lack of labor protection in China.

In this study, the mean time interval between primary cataract surgery and surgical intervention for IOL dislocation was 5.63 years, shorter than the times reported in previous studies (8.04 years [13] and 8.5 years [15]). The reason for this difference may be related to the inclusion of patients with trauma and out-of-the-bag IOL dislocation in this study. As IOL dislocation occurred in more than half of the eyes within 5 years and in less than 9% of the eyes in excess of 10 years, a minimum follow-up of 5–10 years is needed for the patients with IOL dislocation risk factors, even as the primary cataract surgery is uneventful.

The mean age of IOL dislocation patients was 48.10 ± 20.17 years, and 70.4% were male in this study. The majority of the patients were young- and middle-aged males, which is in accord with the findings in our study, in which trauma is listed as the first common risk factor for IOL dislocation. China is the world's largest population, and young- and middle-aged men are the most engaged in heavy labor work that lacks occupational protection, thus representing a significant trauma exposure risk. Blunt force after primary IOL implantation could result in zonular rupture and dialysis and often leads to in-the-bag IOL dislocations, while open-globe injuries before primary IOL implantation often lead to out-of-the-bag dislocations, with capsule rupture or capsule incompleteness.

High myopia was another one of the most common risk factors associated with IOL dislocation in this study. At the same time, we found that AL longer than 30 mm was prone to result in IOL in-the-bag dislocation than out-of-the-bag dislocation, for in-the-bag dislocation patients with AL longer than 30 mm were significantly higher than out-of-the-bag dislocation patients. Part of the reason may be the variability in ethnicity, whereas high myopia has a higher prevalence than pseudoexfoliation in China, which resulted in zonule weaknesses. Longer axial length of the eyeball causes 360-degree stretching and dehiscence of zonular fibers, which fails to adequately resist the tension of the anterior capsule contraction and results in progressive capsular contraction or the zonular dialysis. Twenty-one eyes had anterior capsule contraction. And the most common causes, except for high myopia, are retinitis pigmentosa, chronic uveitis, glaucoma, and others.

Pars plana vitrectomy is the most common surgery associated with IOL dislocation. The most common causes of vitrectomy include retinal detachment (36.1%) and traumatic vitreoretinopathy (31.1%). Vitrectomy for retinal detachment is more likely to cause IOL dislocation than vitrectomy for macular disease (36.1% retinal detachment versus 8.2% macular hole). Part of the reason may be that aggressive peripheral vitrectomy with scleral depression during the vitrectomy for retinal detachment may damage the posterior zonular fibers. Traumatic vitreoretinopathy is often associated with anterior or posterior capsular rupture. Also before 2011, pars plana vitrectomy used a 20-gauge vitrectomy system with a lower vitrector cutting rate (20G vitrectomy 800–2500 cuts per minute versus 23G vitrectomy 2500–7500 cuts per minute) and a larger diameter of vitrectomy probe (20G vitrectomy probe 1.0 mm versus 23G vitrectomy probe 0.5 mm) than a 23-gauge vitrectomy system, which is more likely to damage posterior zonular fibers and the posterior lens capsule. Additionally, damage to the posterior lens capsule may cause traumatic injury of the capsule integrity and, in turn, was linked to a larger percentage of out-of-the-bag dislocations in the present study. A total of 30 eyes with IOL dislocation had previously undergone surgeries for congenital cataract in this study. Because of inadequate primary posterior capsulotomy and/or anterior vitrectomy during the primary IOL implantation, the anterior vitreous face formed a scaffold for the proliferation of lens epithelial cells, which may contribute to IOL shift forward and result in IOL anterior displacement.

The most common surgical techniques to treat IOL dislocation were IOL exchange and IOL refixation. The usual treatment for in-the-bag dislocations were intraocular lens complex explantation with or without new IOL sulcus fixation. However, for out-of-the-bag IOL dislocation, several factors may influence the choice of old IOL refixation or new IOL exchange: the amount of capsule support, the position and degree of the zonular dialysis, and other factors. In this study, we found that 19 eyes were subjected to two IOL repositionings before the latest sutured scleral refixation. Part of the reason is that the IOL is placed in the sulcus; however, the capsular support is inadequate, leading to repeated IOL dislocation, especially downstairs dislocation. The lens capsule may be able to temporarily hold an IOL in the sulcus but not enough for permanent fixation. We suggest that once out-of-the-bag IOL dislocation occurs, the old IOL should be removed, and a new, larger diameter optic IOL should be fixed to the sulcus. The three-piece IOL is not recommended to be used, for the rigid haptics of a three-piece IOL may aggravate further damage to the already weak lens zonule. Moreover, with the advancement of technology, numerous surgical techniques for scleral fixation of dislocated IOL have been developed, for example, an innovative scleral pockets technique for intrascleral fixation of IOL [16], which is conducive to better positioning of IOL.

## 5. Conclusion

In conclusion, the most common IOL dislocation is out-of-the-bag dislocation; the most common risk factors include trauma, high myopia, and eyes undergoing pars plana vitrectomy; a minimum follow-up of 5 years is suggested for eyes predisposed for undergoing cataract surgery.

## Figures and Tables

**Table 1:** Baseline clinical characteristics.

Baseline clinical characteristics	Mean ± SD	Range
Age	48.10 ± 20.17 years	2 to 85 years
Duration of IOL dislocation	5.63 ± 5.13 years	1 day to 23.39 years
Time between IOL dislocation and surgical intervention	0.53 ± 1.40 years	1 day to 15.39 years

Axial length	25.65 ± 3.31 mm	17.74 to 36.69 mm
In-the-bag dislocation	26.51 ± 3.78 mm	20.86 to 35.86 mm
Out-of-the-bag dislocation	25.34 ± 3.07 mm	17.74 to 36.69 mm

**Table 2:** Associated ocular conditions with IOL dislocation.

Associated ocular conditions	Eyes (*n*/328)
Trauma	136 (41.5%)
Open-globe injuries	95 (29.0%)
Before primary IOL implantation	93 (28.4%)
After primary IOL implantation	2 (0.6%)
Ocular blunt trauma	41 (12.5%)
Before primary IOL implantation	6 (1.8%)
After primary IOL implantation	35 (10.7%)

Ocular diseases	172 (52.4%)
High myopia	108 (32.9%)
Lens subluxation	32 (9.8%)
Retinitis pigmentosa	15 (4.6%)
Chronic uveitis	6 (1.8%)
Glaucoma	5 (1.5%)
Iris cyst	4 (1.2%)
Ocular siderosis	2 (0.6%)

Ocular surgeries	108 (32.9%)
Pars plana vitrectomy	61 (18.6%)
Congenital cataract surgery	30 (9.1%)
Trabeculectomy	6 (1.8%)
Strabismus correction	6 (1.8%)
Scleral buckling	3 (0.9%)
Penetration keratoplasty	2 (0.6%)

Capsule rupture	42 (12.8%)
Nd : YAG laser capsulotomy	8 (2.4%)
Capsular contraction syndrome	21 (6.4%)

**Table 3:** Distributions of IOL dislocation based on classification systems.

Classifications of IOL dislocation	Eyes (*n*/328)
Relationship with capsule	
In-the-bag dislocations	85 (25.9%)
Out-of-the-bag dislocations	243 (74.1%)

IOL dislocation position	
In the anterior chamber	39 (11.9%)
In the posterior chamber and/or the anterior vitreous cavity	184 (56.1%)
In deep vitreous cavity/vitreous completely	105 (32.0%)

**Table 4:** Sites and grades of IOL dislocation.

Sites and grades of dislocated IOL	Eyes (*n*/184)
Dislocation grades	
Mild	17 (9.2%)
Moderate	85 (46.2%)
Severe	82 (44.6%)

Dislocation sites	
Superior	3 (1.6%)
Superonasal	5 (2.7%)
Nasal	7 (3.8%)
Inferonasal	25 (13.6%)
Inferior	121 (65.8%)
Inferotemporal	18 (9.8%)
Superotemporal	5 (2.7%)

**Table 5:** Ocular conditions associated with in-the-bag dislocations and out-of-the-bag dislocations.

Associated ocular conditions	In-the-bag dislocation (eyes/85)	Out-of-the-bag dislocation (eyes/243)
Trauma	26 (30.6%)	110 (45.3%)
Ocular blunt trauma	20 (23.5%)	21 (8.6%)
Open-globe injuries	6 (7.1%)	89 (36.6%)
High myopia	36 (42.4%)	72 (29.6%)
Lens subluxation	7 (8.2%)	25 (10.3%)
Retinitis pigmentosa	13 (15.3%)	2 (0.8%)
Chronic uveitis	6 (7.1%)	0 (0%)
Glaucoma	2 (2.4%)	3 (1.2%)
Iris cyst	0 (0%)	4 (1.6%)
Ocular siderosis	1 (1.2%)	1 (0.4%)
Pars plana vitrectomy	11 (12.9%)	50 (20.6%)
Congenital cataract surgery	2 (2.4%)	28 (11.5%)
Strabismus correction	1 (1.2%)	5 (2.1%)
Trabeculectomy	2 (2.4%)	4 (1.6%)
Scleral buckling	1 (1.2%)	2 (0.8%)
Penetration keratoplasty	1 (1.2%)	1 (0.4%)
Capsule rupture	1 (1.2%)	41 (16.9%)
Nd : YAG laser capsulotomy	0 (0%)	8 (3.3%)

**Table 6:** Surgical techniques to correct IOL dislocation.

Surgical techniques	Eyes (*n*/328)
Exchange	131 (39.9%)
Sutured scleral fixation	98 (29.9%)
Iris-claw IOL	12 (3.7%)
Positioning in ciliary sulcus	21 (6.4%)

Reposition	30 (9.1%)
Ciliary sulcus reposition	30 (9.1%)

Refixation	85 (25.9%)
Sutured scleral refixation	78 (23.8%)
Sutureless intrascleral refixation	7 (2.1%)

Explantation	82 (25%)
Simple explantation	34 (10.4%)
Explantation plus anterior vitrectomy	22 (6.7%)
Explantation plus pars plana vitrectomy	26 (7.9%)

## Data Availability

The data used to support the findings of this study are available from the corresponding author upon request.
